# Comparison of guidelines for intraductal papillary mucinous neoplasm: What is the next step beyond the current guidelines?

**DOI:** 10.1002/ags3.12012

**Published:** 2017-06-16

**Authors:** Masafumi Nakamura, Yoshihiro Miyasaka, Yoshihiko Sadakari, Kenjiro Date, Takao Ohtsuka

**Affiliations:** ^1^ Department of Surgery and Oncology Graduate School of Medical Sciences Kyushu University Fukuoka Japan

**Keywords:** guideline, intraductal papillary mucinous neoplasm, nomogram, pancreatic ductal adenocarcinoma, surveillance

## Abstract

Management of intraductal papillary mucinous neoplasm is controversial, and several guidelines have aimed to establish an adequate strategy for surgical resection and surveillance. We compared various intraductal papillary mucinous neoplasm guidelines and considered new matters that are pivotal for improved treatment of intraductal papillary mucinous neoplasm. We identified and compared 11 published guidelines, three of which were major guidelines that mainly referred to the diagnosis and treatment of intraductal papillary mucinous neoplasm (International Association of Pancreatology 2012 guidelines, European Study Group on Cystic Tumours of the Pancreas 2013 guidelines, and American Gastroenterological Association 2015 guidelines). The main concerns of these three guidelines were indication for surgery and follow up of non‐resected lesions. Among the differences between the three guidelines, the period of surveillance recommended was the most controversial matter. Meanwhile, several nomograms have been proposed to improve the diagnosis of intraductal papillary mucinous neoplasm from the level of experts' experiences to that of rational systems. We discuss the adequate strategy of surveillance for intraductal papillary mucinous neoplasm with and without pancreatectomy and nomograms aiming to predict the risk of malignancy in patients with intraductal papillary mucinous neoplasm.

## Abstract 2

In this review, we summarize current available guidelines for IPMN, and discuss concerns that are pivotal for improvement of the guidelines.

## 1 Introduction

Intraductal papillary mucinous neoplasm (IPMN) is a macroscopic precursor lesion of pancreatic cancer and is usually incidentally found during abdominal screening of other diseases. IPMN may progress to colloid carcinoma and tubular carcinoma and accounts for 20% to 30% of pancreatic cancer.^1^, ^2^, ^3^, ^4^ IPMN comprises main duct type (MD‐IPMN), branch duct type (BD‐IPMN), and mixed type (MD + BD). MD‐IPMN more frequently progresses to malignant lesions (60‐70%)^2^, ^5^, ^6^, ^7^ than BD‐IPMN,^2^, ^5^, ^8^ although BD‐IPMN is more commonly seen than MD‐IPMN.^9^, ^10^ Researchers have also hypothesized that benign BD‐IPMN may develop distinct pancreatic ductal adenocarcinoma (PDAC) synchronously or metachronously. The natural history and high incidence of BD‐IPMN make its surveillance controversial. Hence, several guidelines have been developed with an aim to establish an adequate strategy for surgical resection and surveillance of IPMN. We identified 11 available guidelines and further compared three major guidelines of IPMN published by the International Association of Pancreatology in 2012 (IAP2012),^11^ European Study Group on Cystic Tumours of the Pancreas in 2013 (EURO),^12^ and American Gastroenterological Association in 2015 (AGA).^13^ We also herein present a discussion of new topics that are pivotal for the next step in improving the surveillance and treatment of IPMN.

## 2 Comparison of Current Guidelines for IPMN

Table 1 shows 11 published guidelines concerning IPMN. Although they include other pancreatic lesions such as cystic neoplasms or pancreatic intraepithelial neoplasia, most of them focus mainly on management of IPMN. Among them, three guidelines deal with pathological issues (#1, #10, #11).^14^, ^15^, ^16^ An illustrated consensus (#1) proposed a pathological definition of IPMN for differentiation from pancreatic intraepithelial neoplasia.^16^ A revised classification system and recommendations (#10) used the two‐tier grading system (low‐grade/high‐grade) instead of the three‐tier grading system (low‐grade/intermediate‐grade/high‐grade) proposed by the Armed Forces Institutes of Pathology.^15^, ^17^ The Recommendation of Verona consensus meeting (#11) provided the principles of pathological evaluation and reporting of IPMN.^14^

**Table 1:** Published guidelines concerning intraductal papillary mucinous neoplasm

	Author	Year	Title	Journal
#1	Hruban et al.^16^	2004	An illustrated consensus on the classification of pancreatic intraepithelial neoplasia and intraductal papillary mucinous neoplasms	Am J Surg Pathol
#2	Jacobson et al.^18^	2005	ASGE guideline: The role of endoscopy in the diagnosis and the management of cystic lesions and inflammatory fluid collections of the pancreas	Gastrointest Endosc
#3	Tanaka et al.^19^	2006	International consensus guidelines for management of intraductal papillary mucinous neoplasms and mucinous cystic neoplasms of the pancreas	Pancreatology
#4	Society for Surgery of the Alimentary Tract^20^	2007	SSAT patient care guidelines. Cystic neoplasms of the pancreas	J Gastrointest Surg
#5	Tanaka et al.^11^	2012	International consensus guidelines 2012 for the management of IPMN and MCN of the pancreas	Pancreatology
#6	Canto et al.^21^	2013	International Cancer of the Pancreas Screening (CAPS) Consortium summit on the management of patients with increased risk for familial pancreatic cancer	Gut
#7	Del Chiaro et al.^12^	2013	European experts consensus statement on cystic tumours of the pancreas	Dig Liver Dis
#8	Buscarini et al.^22^	2014	Italian consensus guidelines for the diagnostic work‐up and follow‐up cystic pancreatic neoplasms	Dig Liver Dis
#9	Vege et al.^13^	2015	American Gastroenterological Association Institute Guideline on the Diagnosis and Management of Asymptomatic Neoplastic Pancreatic Cysts	Gastroenterology
#10	Basturk et al.^15^	2015	A revised classification system and recommendations from the Baltimore consensus meeting for neoplastic precursor lesions in the pancreas	Am J Surg Pathol
#11	Adsay et al.^14^	2016	Pathologic evaluation and reporting of intraductal papillary mucinous neoplasms of the pancreas and other tumoral intraepithelial neoplasms of pancreatobiliary tract: recommendations of Verona consensus meeting	Ann Surg

The American Society for Gastrointestinal Endoscopy guideline (#2) discussed the use of endoscopic modalities, such as endoscopic ultrasonography (EUS), EUS‐guided fine‐needle aspiration (EUS‐FNA), and endoscopic retrograde cholangiopancreatography (ERCP), in differentiating IPMN from other cystic pancreatic lesions.^18^ In 2006, international consensus guidelines (the IAP2006) (#3) were the first comprehensive guidelines referring to the diagnosis, indications for resection, and surveillance of IPMN.^19^ The Society for Surgery of the Alimentary Tract Patient Care Guidelines (#4) provided general information on the categories, symptoms, diagnosis, and treatment of cystic neoplasms of the pancreas; however, these guidelines were unable to indicate either definite criteria for surgical resection or a surveillance strategy.^20^ The consensus statements of the International Cancer of the Pancreas Screening consortium summit (#6) proposed that IPMN was a potential target for early detection and treatment in individuals at high risk for pancreatic cancer.^21^ Italian consensus guidelines (#8) mainly focused on the diagnostic and follow‐up strategies of pancreatic cystic neoplasms.^22^

The remaining three guidelines (#5, #7, #9) are the current comprehensive guidelines citing diagnostic work‐up, indications for surgery, surveillance after surgery, and surveillance of non‐resected IPMN.^11^, ^12^, ^13^ In this section, we compare these three guidelines (Table 2). The IAP2012 (#5) and EURO (#7) guidelines are expert consensuses, whereas the AGA guidelines (#9) were established by an evidence‐based approach using the GRADE (Grading of Recommendations Assessment, Development and Evaluation) framework and the PICO (Patient problem or population, Intervention, Comparison and Outcome) format. However, the AGA guidelines noted that all evidence concerning the management of pancreatic cystic neoplasms was of very low quality because most of the available data were from retrospective case series.

**Table 2:** Comparison of the guidelines citing diagnostic work‐up, indications for surgery, surveillance after surgery, and surveillance of non‐resected IPMN

	International consensus guidelines 2012 (IAP2012)^11^	European Experts Consensus Statement (EURO)^12^	American Gastroenterological Association Institution Guideline (AGA)^13^
Types of guideline	Consensus guidelines	Consensus guidelines	Evidence‐based guidelines
Type of tumor/neoplasm	IPMN and MCN	Cystic tumor of the pancreas	Asymptomatic neoplastic pancreatic cyst
Initial assessment			
Radiological assessment	CT and/or MRI	CT and/or MRI	MRI
EUS (EUS‐FNA)	Recommended if worrisome features (cyst ≥3 cm, thickened cyst wall, MPD 5‐9 mm, non‐enhancing nodule, caliber change of MPD, lymphadenopathy) are present	EUS is useful for surgical indication, and EUS‐FNA is useful for differential diagnosis	EUS‐FNA is recommended if two or more high‐risk features (cyst ≥3 cm, dilated MPD, solid component) are present
Indication for resection	High‐risk stigmata (obstructive jaundice, enhanced nodule, MPD ≥10 mm) or worrisome features + significant EUS findings (definite nodule, MPD involvement, positive cytology)	Absolute indications (cyst ≥4 cm, symptoms, mural nodules, MPD ≥6 mm), relative indications (rapid size increase, serum CA19‐9 elevation)	Two or more high‐risk features + positive cytology in EUS‐FNA
Surveillance of non‐resected cases	<1 cm: CT/MRI every 2‐3 years; 1‐2 cm: CT/MRI every 12 months; 2‐3 cm: EUS every 3‐6 months; >3 cm: close surveillance alternating MRI with EUS every 3‐6 months	Surveillance with MRI (or EUS) 1st year: every 6 months; 2nd‐5th year: every 12 months; >5th year: every 6 months	1st, 3rd, 5th year: MRI. No more surveillance if no significant change has been recognized
Surveillance of resected cases	Invasive IPMN: same surveillance as PDAC; noninvasive IPMN (without residual lesion): repeat examination at 2 and 5 years	Invasive IPMN: same surveillance as PDAC; noninvasive IPMN: MRI or EUS every 12 months	Cyst with invasive cancer or dysplasia: MRI every 2 years; cyst without high‐grade dysplasia or malignancy: no routine surveillance

CT, computed tomography; EUS, endoscopic ultrasonography; EUS‐FNA, endoscopic ultrasound‐guided fine‐needle aspiration; IPMN, intraductal papillary mucinous neoplasm; MCN, mucinous cystic neoplasm; MPD, main pancreatic duct; MRI, magnetic resonance imaging; PDAC, pancreatic ductal adenocarcinoma.

Comparison of these three guidelines shows that the main concerns are indications for surgery and follow up of non‐resected lesions. Because IPMN varies from low‐grade dysplasia to invasive cancer, it is important to establish a reliable indication that can predict malignant lesions. In the IAP2012 guidelines, high‐risk stigmata (obstructive jaundice, an enhanced nodule, and a main pancreatic duct (MPD) of ≥10 mm) are absolute indications for surgery. If worrisome features are recognized (cysts ≥3 cm, thickened cyst wall, MPD size 5‐9 mm, non‐enhancing nodules, caliber change of pancreatic duct with pancreatic atrophy, or lymphadenopathy), EUS is recommended. Surgery should be considered if a definite nodule, MPD involvement, or positive cytology is seen on EUS. In the EURO guidelines, absolute indications for surgery include a cyst diameter ≥4 cm, pancreas‐related symptoms (jaundice, diabetes, or acute pancreatitis), mural nodules, and MPD >6 mm; relative indications are a rapid increase in size and an elevated serum CA19‐9 concentration. In the AGA guidelines, indications for surgery are positive cytology on EUS‐FNA which is carried out if at least two of three high‐risk features are present (cyst ≥3 cm, dilated MPD, or solid component). Threshold of the AGA guidelines was set at a high level from the viewpoint of medical economics. Therefore, the AGA guidelines carry a higher risk of excluding resection of malignant lesions than the other two guidelines. Lekkerkerker et al.^23^ carried out a comparative analysis of the three guidelines using 75 cases of IPMN and reported that 12% of malignant lesions would have been overlooked with the AGA guidelines whereas none would have been missed with the IAP2012 or the Euro guidelines. In addition, Singhi et al.^24^ analyzed 225 patients who underwent EUS‐FNA for pancreatic cystic lesions and pointed out that the AGA guidelines would have missed 45% of malignant IPMN lesions.

In the IAP2012 guidelines, surveillance of non‐resected IPMN depends on the size of the cyst (<1 cm: CT/MRI every 2‐3 years; 1‐2 cm: CT/MRI every 12 months in the first 2 years, then at longer intervals; 2‐3 cm: EUS every 3‐6 months, then at longer intervals alternating MRI with EUS; and >3 cm: close surveillance alternating MRI with EUS every 3‐6 months). The IAP2012 guidelines do not indicate a definite period of surveillance, although they state that “there are no good long‐term data to indicate whether surveillance can be safely discontinued after long‐term stability.” In the EURO guidelines, surveillance of non‐resected IPMN should be carried out every 6 months in the first year, and annual surveillance with MRI (or EUS) is recommended for the following 5 years. After the fifth year, surveillance is recommended every 6 months. The EURO guidelines also state that surveillance should be continued as long as the patient is fit for surgery. The AGA guidelines suggest that patients with non‐resected IPMN should undergo surveillance with MRI at 1, 3, and 5 years and that surveillance can be discontinued if no significant change is recognized. The matter of termination of surveillance of non‐resected IPMN will be discussed in another section.

The IAP2012 and EURO guidelines mention the method of IPMN resection. Both guidelines agree that oncological resection should be carried out if malignancy is suspected and that partial resection is preferred to total pancreatectomy in the treatment of multiple BD‐IPMN. However, the guidelines disagree regarding the management of dysplasia at the margin of intraoperative frozen sections. In the IAP2012 guidelines, additional resection is recommended if high‐grade dysplasia or invasive carcinoma is recognized at the margin, and the presence of low‐/moderate‐grade dysplasia does not require further treatment. The EURO guidelines recommend additional resection even if moderate‐grade dysplasia is recognized at the margin.

Another point is surveillance after IPMN resection. For invasive IPMN, the IAP2012 and EURO guidelines recommend the same surveillance as carried out for pancreatic cancer. In contrast, the AGA guidelines suggest MRI surveillance every 2 years even after invasive IPMN. For noninvasive IPMN, the IAP2012 guidelines recommend repeat examinations at 2 and 5 years for new recurrences after resection, whereas they also suggest a 6‐month interval, which is appropriate for surveillance considering the risk of PDAC development. The EURO guidelines recommend annual follow up for noninvasive IPMN. The AGA guidelines suggest MRI surveillance every 2 years after resection of high‐grade IPMN, but they do not recommend routine surveillance after resection of pancreatic cysts without high‐grade dysplasia or invasive malignancy. This matter will also be discussed later.

## 3 Prediction of Malignant IPMN by Nomogram

Besides expert opinion‐based guidelines, several efforts have been made to establish a nomogram as a more rational system with which to predict malignant IPMN, as listed in Table 3.^8^, ^25^, ^26^, ^27^, ^28^, ^29^ Shimizu et al.^27^ first reported a nomogram for IPMN, which covered both MD‐IPMN and BD‐IPMN simultaneously. Jang et al.^8^ included the largest clinicopathological data set of 645 patients and focused exclusively on BD‐IPMN. Correa‐Gallego et al.^25^ and Attiyeh et al.^26^ devised two independent nomograms for MD‐IPMN and BD‐IPMN, respectively. In these three nomograms, which contain an independent nomogram exclusively for BD‐IPMN, cyst size and existence of a solid component are common predictors of malignant lesions in BD‐IPMN,^8^, ^25^, ^26^ and older age is also a predictor in two nomograms.^8^, ^26^ Gemenetzis et al.^29^ included both MD‐IPMN and BD‐IPMN in a nomogram, and one of its factors predicting invasive cancer is the neutrophil‐to‐lymphocyte ratio, which has been reported to be a predictor of invasive cancer and poor prognosis in patients with various types of tumors.^30^, ^31^, ^32^, ^33^

**Table 3:** Discrimination and validation of established nomograms for IPMN

Author	Year	No. patients (training/validation)	Objective IPMN	Intended pathological grade	Selected variables^a^	Validation	C‐index (training/validation)	AUC (training/validation)
Correa‐Gallego et al.^25^	2013	123/123	BD (& Mix/MD)	High‐grade dysplasia and invasive carcinoma	Solid component (Y/N)	Internal validation	0.74/0.74	
					Lesion diameter (cm)			
					Weight loss (Y/N)			
Attiyeh et al.^26^	2016	402/172	BD (& Mix/MD)	High‐grade dysplasia and invasive carcinoma	Solid component (Y/N)	External validation	0.82/0.81	
					Lesion diameter (>3.0 cm)			
					Age			
					Gender male (Y/N)			
					Symptomatic (Y/N)			
Jang et al.^8^	2016	645/624	BD	High‐grade dysplasia and invasive carcinoma	Solid component (Y/N)	External validation		0.783/0.737
					Lesion diameter (cm)			
					Age			
					MPD diameter (mm)			
					Serum CEA (ng/mL)			
					Serum CA19‐9 (U/mL)			
Shimizu et al.^27^, ^28^	2010	81/180	All types of IPMN	High‐grade dysplasia and invasive carcinoma	Size of nodules (mm)	External validation		0.903/0.760
	2015				Type of lesion (MD‐IPMN)			
					Gender female (Y/N)			
					Cytology			
Gemenetzis et al.^29^	2016	272/‐	All types of IPMN	Invasive carcinoma	Solid component (Y/N)	No validation	0.895/‐	
					Lesion diameter (cm)			
					MPD dilatation >5 mm			
					Jaundice (Y/N)			
					NLR			

AUC, area under the curve; BD‐IPMN, branch duct‐type intraductal papillary mucinous neoplasm; C‐index, concordance‐index; CA19‐9, carbohydrate antigen 19‐9; CEA, carcinoembryonic antigen; MD‐IPMN, main duct‐type IPMN; Mix, MD + BD‐IPMN; MPD, main pancreatic duct; NLR, neutrophil‐to‐lymphocyte ratio.

a Selected variables in two nomograms established by Correa‐Gallego et al.^25^ and Attiyeh et al.^26^ are listed in the nomogram targeting BD‐IPMN.

These nomograms were validated by two methods: internal validation and external validation (Table 3). External evaluation of the nomogram is recommended because of objectivity and repeatability.^34^ Three studies assessed their nomograms by external validation,^8^, ^26^, ^28^ and one study used internal validation,^25^ and one used no validation.^29^ The concordance index and area under the curve derived from validation are used to estimate validity of the nomogram. External validation of the nomogram as established by Attiyeh et al.^26^ demonstrated good discrimination power (concordance index in the training and validation sets was 0.82 and 0.81, respectively).

A nomogram that simultaneously covers both BD‐IPMN and MD‐IPMN is feasible and versatile because morphological classification is sometimes vague. However, validation results have demonstrated that nomograms limited to BD‐IPMN are precise with good discrimination. The nomogram discrimination power depends on patient volume and accurate patient data. Therefore, in the near future, establishment of a more convenient and reliable nomogram with which to predict malignant IPMN with greater accumulation of clinical data is expected.

## 4 Surveillance after Resection of IPMN

The aim of surveillance after resection of IPMN is early detection of recurrence. “Recurrence” after resection of IPMN includes several situations: recurrence of associated carcinoma, development of a new IPMN lesion, and progression of unresected IPMN in the remnant pancreas. Besides these types of IPMN recurrence, PDAC distinct from IPMN may also develop in the remnant pancreas. In this section, recurrence of carcinoma derived from a resected IPMN is designated as a recurrent lesion, and newly developing or progressing IPMN and newly developing PDAC in the remnant pancreas are designated as remnant pancreatic lesions.

Recurrent lesions frequently develop and influence patient survival after resection of invasive IPMN. Disease‐specific survival of patients with recurrent lesions remains low.^35^, ^36^, ^37^, ^38^ Therefore, it is reasonable to carry out postoperative surveillance for invasive IPMN as done for PDAC. Besides recurrence of invasive IPMN, several investigators have reported recurrent lesions after resection of noninvasive IPMN.^39^, ^40^, ^41^, ^42^, ^43^, ^44^ We also experienced peritoneal dissemination after resection of high‐grade IPMN.^7^ Even after resection of noninvasive IPMN, especially high‐grade IPMN, attention should be paid to the development of recurrent lesions.

Several follow‐up studies have revealed frequent development of remnant pancreatic lesions after resection of IPMN^41^, ^45^, ^46^, ^47^, ^48^, ^49^, ^50^ (Table 4). He et al.^46^ reported that the 10‐year incidences of new IPMN, new IPMN requiring surgery, and invasive carcinoma in the remnant pancreas after resection of noninvasive IPMN were 62%, 18%, and 38%, respectively. Hirono et al.^41^ documented 14 remnant pancreatic lesions in 257 patients who underwent IPMN resection. Among these 14 remnant pancreatic lesions, five (35.7%) developed more than 5 years after the initial operation. We reported that the 5‐ and 10‐year cumulative incidences of high‐risk lesions, including high‐grade and invasive IPMN and PDAC, were 7.8% and 11.8%, respectively.^48^ These data indicate that long‐term surveillance (as long as the patient is fit for surgery) is required to detect remnant pancreatic lesions.

**Table 4:** Reports of remnant pancreatic lesions

Author	Year	No. total patients	No. noninvasive IPMN patients	Follow‐up period	Remnant pancreatic lesion		Five‐ and 10‐ year cumulative incidences of remnant pancreatic lesions
					No. total patients	No. patients with malignant lesions	
Schnelldorfer et al.^50^	2008	208	145	3.2 years^a^	11	3	NA
Miller et al.^47^	2011	243	243	73 months^a^	31 (+ 38^c^)	4 (invasive IPMN)	NA
Moriya & Traverso^49^	2012	203	160	40 months^b^	17 (+ 14^c^)	4	NA
He et al.^46^	2013	130	130	38 months^b^	22	5 (invasive lesion)	7% and 38%^d^
Frankel et al.^45^	2013	192	192	46 months^b^	40	3	NA
Hirono et al.^41^	2016	257	172	53.5 months^b^	14	12 (IPMN 7/PDAC 5)	NA
Miyasaka et al.^48^	2016	195	160	47 months^b^	29	13 (IPMN 6/PDAC 7)	7.8% and 11.8%^e^

a Mean.

b Median.

c Residual lesions.

d Invasive cancer.

e High‐risk lesions.

IPMN, intraductal papillary mucinous neoplasm; NA, not available; PDAC, pancreatic ductal adenocarcinoma.

Hirono et al.^41^ reported that a candidate risk factor for these remnant pancreatic lesions was dysplasia at the pancreatic cut margin, which included not only malignant lesions but also low‐grade lesions. Pea et al.^51^ analyzed 260 noninvasive IPMN and concluded that a family history of pancreatic cancer and high‐grade IPMN as the initial lesion were risk factors for malignant lesions in the remnant pancreas. Our analysis revealed that high‐grade/invasive IPMN, MD‐IPMN, and an IPMN located in the distal pancreas as the initial lesion were risk factors for development of high‐grade/invasive IPMN in the remnant pancreas. Presence of concomitant PDAC as the initial lesion was a risk factor for development of PDAC in the remnant pancreas.^48^ It has been suggested that a postoperative surveillance strategy designed according to the degree of dysplasia may be reasonable for noninvasive IPMN, although surveillance of the remnant pancreas after resection of low‐grade BD‐IPMN is still necessary for metachronous development of PDAC.^11^

## 5 Surveillance for IPMN without Pancreatectomy

Although several guidelines have provided recommendations regarding management of IPMN without pancreatectomy,^11^, ^12^, ^13^, ^19^ an adequate surveillance protocol has not yet been established. Whereas BD‐IPMN infrequently develop invasive carcinoma, the incidence of PDAC concomitant with BD‐IPMN (4.5‐8.3%) is reportedly higher than that of malignant transformation of BD‐IPMN (0.0‐3.0%)^52^, ^53^, ^54^, ^55^ (Table 5). Thus, attention should be paid not only to malignant transformation of IPMN itself, but also to the development of concomitant PDAC during surveillance for BD‐IPMN. In addition, surveillance carried out at shorter intervals might be needed to detect concomitant PDAC because Uehara et al.^54^ reported that most concomitant PDAC were discovered at an advanced stage or in an unresectable condition during their surveillance of IPMN despite carrying out surveillance every 3‐6 months.

**Table 5:** Reports of surveillance of IPMN without pancreatectomy

Author	Year	No. total patients	Follow‐up period	No. patients with malignant lesions	No. patients with concomitant PDAC	Cumulative incidence of concomitant PDAC
Tanno et al.^52^	2008	82	61 months^b^	1	NA	NA
Uehara et al.^54^	2008	60	87 months^a^	2	5	5 years: 6.9%
Tanno et al.^55^	2010	89	64 months^b^	0	4	5 years: 3.0%; 10 years: 8.8%
Kamata et al.^53^	2014	102	42 months^b^	0	7	3 years: 4.0%; 5 years: 8.8%

a Mean.

b Median.

IPMN, intraductal papillary mucinous neoplasm; NA, not available; PDAC, pancreatic ductal adenocarcinoma.

Another important issue is how long the surveillance should be continued in terms of detecting PDAC concomitant with IPMN. The AGA guidelines suggest that patients without significant changes in their pancreatic cyst for 5 years can discontinue surveillance. However, in their long‐term surveillance of BD‐IPMN without pancreatectomy, Tanno et al.^55^ reported that the 5‐ and 10‐year cumulative incidences of the development of PDAC distinct from BD‐IPMN were 3.0% and 8.8%, respectively. These findings suggest that surveillance of IPMN should be continued for more than 5 years even when the IPMN shows no significant morphological change.

Considering the high incidence of IPMN, the estimated prevalence of which is approximately 26/100 000 and increases three‐ to fourfold in individuals older than 60 years,^56^, ^57^ determination of patients at high risk of developing distinct PDAC is urgently needed. One possible risk factor is a family history of PDAC. Nehra et al.^58^ showed that concurrent PDAC in patients who underwent resection of IPMN were more commonly observed in those with than in those without a family history of PDAC (11.1% vs 2.9%, respectively; *P*=.02). Meanwhile, Mandai et al.^59^ showed that the frequency of concomitant PDAC in patients with BD‐IPMN or mixed IPMN was significantly higher in patients with than in those without a family history; however, median age of the patients with a family history was significantly higher than in those without a family history. To exclude the influence of age, Mandai et al. carried out another comparison in which they limited the age of patients to ≥70 years and, as a result, the frequency of concomitant PDAC was not significantly different between these two groups.

Although several Japanese investigators^2^, ^53^ have recommended carrying out surveillance every 6 months for detection of PDAC concomitant with IPMN, the diagnostic modality that should be included in this protocol remains unclear. Alternating CT and MRCP twice a year seems to be a popular protocol in Japan,^60^ but it often fails to diagnose resectable PDAC.^61^ Conversely, Kamata et al.^53^ prospectively surveyed 167 patients with untreated IPMN using an EUS‐based protocol and found seven metachronous developments of concomitant PDAC, all of which could be treated by resection. Moreover, the diagnostic ability of EUS to detect these resectable concomitant PDAC when carried out twice a year was superior to other imaging modalities such as percutaneous ultrasonography, CT, or MRI. However, EUS seems to be invasive, and the diagnostic ability of EUS to detect early‐stage concomitant PDAC largely depends on the operator's skill; thus, EUS cannot be carried out at the same level globally. Other Japanese investigators^62^, ^63^ have demonstrated the important roles of pancreatic juice cytology under ERCP to diagnose stage 0 or I PDAC, although ERCP is still associated with a risk of pancreatitis and is therefore unsuitable for screening. Nevertheless, establishment of a surveillance protocol using an adequate combination of EUS/ERCP for high‐risk patients would contribute to an increase in the number of patients who are diagnosed with resectable concomitant PDAC.

In summary, it might be important to carry out long‐term surveillance at short intervals for more than 5 years as long as the patient is fit for surgery. A common protocol for IPMN with and without resection is alternate CT and MRCP (EUS) twice a year in Japan;^60^ however, its ability to improve overall survival and its cost‐effectiveness should be evaluated. Further investigation using a prospective protocol with a large number of patients is needed to clarify the precise incidence of concomitant PDAC distinct from IPMN, to establish the optimal interval and period of surveillance, and to determine the most reliable risk factors for concomitant PDAC.

## Disclosure

Conflict of Interest: Authors declare no conflicts of interest for this article.
